# The Bank of Standardized Stimuli (BOSS), a New Set of 480 Normative Photos of Objects to Be Used as Visual Stimuli in Cognitive Research

**DOI:** 10.1371/journal.pone.0010773

**Published:** 2010-05-24

**Authors:** Mathieu B. Brodeur, Emmanuelle Dionne-Dostie, Tina Montreuil, Martin Lepage

**Affiliations:** 1 Douglas Mental Health University Institute and Department of Psychiatry, McGill University, Montreal, Quebec, Canada; 2 Department of Psychology, Université de Montréal, Montreal, Quebec, Canada; 3 Department of Psychology, Université du Québec à Montréal, Montreal, Quebec, Canada; University of Leuven, Belgium

## Abstract

There are currently stimuli with published norms available to study several psychological aspects of language and visual cognitions. Norms represent valuable information that can be used as experimental variables or systematically controlled to limit their potential influence on another experimental manipulation. The present work proposes 480 photo stimuli that have been normalized for name, category, familiarity, visual complexity, object agreement, viewpoint agreement, and manipulability. Stimuli are also available in grayscale, blurred, scrambled, and line-drawn version. This set of objects, the Bank Of Standardized Stimuli (BOSS), was created specifically to meet the needs of scientists in cognition, vision and psycholinguistics who work with photo stimuli.

## Introduction

Experimental stimuli such as visual objects and sounds are essential tools for exploring central processes such as memory, attention, language, etc. They can vary in their perceptual saliency, shape, familiarity, and meaningfulness. Several sets of stimuli have been built and normalized to allow better control over the stimulus features that influence task performance. For instance, there are several databases of words available, such as the Oxford Psycholinguistics database [1]. The words' frequency of use and number of letters have been measured and several variables have been normalized such as the familiarity, meaningfulness, imageability, and concreteness (e.g., [2]). Because they have access to such normative databases, scientists in psycholinguistics can now systematically balance these words' variables across experimental conditions. This control is essential since these variables can modulate behavioral performances and physiological activities in various cognitive tasks [3], [4]. Today, it is inconceivable to conduct a psycholinguistics experiment with sets of stimuli that are not normative.

### Normative datasets of line-drawn pictures

In 1980, Snodgrass and Vanderwart [5] proposed 260 black-and-white line-drawn pictures depicting mostly objects but also animals, vehicles, body parts, and symbolic representations. These pictures were normalized by asking subjects to name the pictures and to rate the familiarity, the visual complexity, and the degree to which the picture matched the image they mentally generated after reading its name. These pictures were rapidly disseminated across the scientific community and became some of the most widely used visual stimuli in cognitive science. This work was pursued in several ways. First, the number of pictures, 260, was increased to 400 by the addition of stimuli from Cycowicz, Friedman, Rothstein and Snodgrass [6] and from the Peabody Picture Vocabulary Test-Revised (PPVT-R) of Dunn and Dunn [7], [8]. This set was also complemented with 299 pictures by Bonin, Peereman, Malardier, Meot, and Chalard [9], 137 pictures by Alvarez and Cuetos [10] and 99 pictures by Nishimoto, Miyawaki, Ueda, Une, and Takahashi [11]. Other normative sets of pictures of objects, proposed by Dell'Acqua, Lotto and Job [12], Kremin and colleagues [13], and Masterson and Druks [14] are also available as well as sets of pictures depicting actions [14], [15]. To these, one can also add older sets of pictures (e.g., a set from the Max Planck Institute for Psycholinguistics and the Abbate and LaChapelle [16]) that have recently been normalized [17], [18], [19]. Finally, there are sets made from modified versions of the Snodgrass and Vanderwart's pictures. These modified pictures include grayscale and colored versions [20], chimeric objects [21], [22], rotated objects [23], [24], silhouettes [25], [26], straight-line versions of objects, fragmented pictures [27], and degraded pictures [25]. Most of these modifications reduce stimulus information and can thus be used for tasks testing very specific visual processing aspects involved in identification processes. For instance, De Winter and Wagemans [28] used silhouettes, degraded, fragmented, and straight-line versions of pictures to examine aspects of contour-based object identification and segmentation.

A second line of work consisted in collecting norms from different populations. Norms were examined in children to study the developmental characteristics of picture and naming processing [6], [7], [29], [30], [31], [32]. Overall, it was found that children named most of the pictures like adults but the alternative names were greater, more various and tended to be shorter [6], [7], [29]. Name agreement was lower in children, particularly the youngest groups, and they were more frequently unable to recognize the object [6], [29], [31]. Finally, familiarity was lower in children [6] but most of the correlations between norms that were observed in adults were also observed in children [6], [7], [30].

Normalization was also collected in different cultural and linguistic populations. This work was indispensable as the norms collected in one country are not necessarily culturally and linguistically adaptable to different populations. For instance, because they are highly unfamiliar in France, indigenous objects to the United States, such as a football helmet, are sometimes removed from stimulus sets [20], [33]. Normalization has been carried out in Chinese [34], [35], Japanese [11], French [9], [15], [33], French Canadian [36], Spanish [37], [38], [39], Portuguese [29], [40], [41], Italian [12], [21], [42], Belgian Dutch [43], Icelandic [44], British English [45], [46], [47], and in many different languages (including German, Bulgarian, Hungrian, Russian, and Swedish) across the same study [13], [17]. Comparisons between the norms from different countries help to better understand how culture and language (including word-particular features) influence the naming behavior as well as other normative variables. With the exception of a few studies (e.g., [35], [38]), it was found that familiarity and visual complexity yielded cross-linguistic correlations, thus suggesting that these variables are weakly affected by cultural differences (see [41] for a review). Correlations are, however, considerably reduced for variables related to the name such as name agreement and image agreement [11], [33], [39], [41]. This was also true for subjects sharing the same language but living in different countries [36]. Looking at the norms for individual pictures reveals that cross-linguistic differences pertain mostly to some pictures that are systematically misidentified or unidentified in some cultures [29], [35]. Despite the cross-linguistic differences, the correlational pattern of results across the norms is generally repeated across studies.

The elaboration of new norms represents a third line of work. The most recent norms were mostly concerned with the names and were thus indirectly related to the picture (age of acquisition of the word, frequency of the word, number of images that come to mind when presented with the word). There are, however, variables directly related to the pictures whose systematic normalization would also be of great interest in many fields of research. This is the case for the manipulability of objects, that is, the quality of an object that the hands control. This variable deserves attention as it is at the center of a well-known brain system view taking into account the object's position and the action that can be applied to the object [48], [49]. Special attention to manipulability is also justified by the fact that non-manipulable objects are named faster than manipulable objects after being controlled for familiarity [50]. Other object features affect behavioral performance and should thus also be normalized. This is the case for some categories of objects known to be processed differently. For instance, although they are likely caused by modality-specific characteristics that differ across categories, non-living objects are more easily named than living objects [51], [52], [53]. Therefore, categories can be predictive of the behavioral performance.

### Normative datasets of photo stimuli

The above-described normative sets of pictures have considerably shaped research in cognitive psychology but although they are still widely used, they cannot fulfill the needs in research requiring photo stimuli. Photos stimuli and line-drawings are characterized by different features that necessarily influence object processing in different ways. Pictures of the Snodgrass and Vanderwart's set are prototypical schematic representations used to evoke a concept. Pictorial features that are not essential to the recognition of the objects are essentially removed [54]. Conversely, photo stimuli come with color, texture, and 3D cues (e.g., shade). These variables can influence the recognition and naming of the object. For instance, it is known that pictures with surface features are named more quickly than those without. This difference was observed between colored and grayscale photos [54] but also between Snodgrass and Vanderwart's original pictures and colored versions of these pictures [20], [55], as well as between line-drawn pictures and colored photos [56], [57]. Moreover, adding textures and lines to drawings [56] and photographic details to photos [58] speeds the naming of these stimuli. The degree of line details in drawings has also been found to change the norms [7]. However, details in objects can also reasonably create the opposite effect and slow down the recognition and naming processes. In real objects, details, as well as objects' design, are not all relevant and can sometimes generate several ambiguities. Photographing only prototypal objects could prevent these ambiguities, but finding prototypal real objects is very unlikely considering the wide variety of objects' designs.

Photo stimuli nevertheless remain incontrovertible stimuli, especially for scientists interested in creating conditions that are as close as possible to real life situations. The importance of these stimuli is reflected in their increased use in recent years, particularly for research on object perception, context processing, and viewpoints. Digital photography and accessibility to imaging software have facilitated the creation of photo stimuli but the normalization of these stimuli is only beginning. There are several sets of normative photo stimuli available to be used as experimental material. One is proposed by Fiez and Tranel [59] and includes photos depicting actions normalized in English as well as in French [60]. There is also the International Affective Picture System (IAPS) [61] distributed by the National Institute of Mental Health. This set includes 480 complex scenes, each assessed on a 1–9 point scale for several dimensions including pleasure, arousal and dominance. It is currently the most widely used set of visual stimuli in the field of research on emotions and anxiety. Several sets of pictures of faces also exist. The two most employed are the Ekman and Friesen [62] and the Karolinska Directed Emotional Face set [63], which includes pictures of individuals depicting various emotional expressions. These sets have been normalized with regards to affective features and, to some extent, on some physical properties. However, given their particular characteristics, their potential use for non-emotion related studies is quite limited. There are nevertheless sets of face pictures normalized for variables not related to emotions but related to identity. For instance, Bonin, Perret, Méot, Ferrand, and Mermillod [64] recently proposed a set of famous faces normalized for name agreement and face agreement.

To our knowledge, the first normative dataset of photos of objects was made by Viggiano and colleagues [65] in 2004. It proposed 174 colored photos of objects and normalized them according to name, familiarity, and visual complexity. They presented these photos as an alternative set of stimuli providing the ecological value that is lacking in the Snodgrass and Vanderwart set. This work represents a very interesting first step but could benefit from expanding work. In addition to the fact that photos were downloaded from the web, 174 objects may sometimes be insufficient for many experimental designs. Moreover, Viggiano and colleagues [65] collected only a limited number of norms. As mentioned earlier, photos provide richer information than line-drawings and this necessarily comes with more variables to normalize. A second set of 147 normative photos, the Hatfield Image Test (HIT), has recently been proposed by Adlington, Laws, and Gale [66]. More norms were collected, including color diagnosticity and age of acquisition but many of the objects were rare (e.g., poncho, honeysuckle, pagoda, armadillo). Rarity was implemented to address the problem of ceiling-level naming performance. These stimuli can therefore be interesting to further study the naming process, but might not be suitable for tasks requiring recognizable objects. Moreover, as with the Viggiano and colleagues set, the number of stimuli is low.

### The goal of the present study

The present work aims at collecting a large sample of photos depicting common objects and normalizing many of these photos according to seven variables. The Bank Of Standardized Stimuli (BOSS) includes 480 normative photos of objects. The tested norms were those of Snodgrass and Vanderwart [5], in addition to the category and the manipulability. Image agreement has also been divided into two more specific variables: object agreement (i.e., the extent to which the object is similar to the one imagined by the subject) and viewpoint agreement (i.e., the extent to which the object is in the position imagined by the subject). Stimuli were also identified as belonging either to living or non-living things. This set of object photos was created specifically to satisfy the needs of many scientists in cognition, vision and psycholinguistics. It can be obtained by contacting the corresponding author.

## Materials and Methods

### Subjects

Seventy-two subjects were recruited through ads published in journals and newspapers, and via online classifieds such as Craigslist. Subjects included people ranging between 17 and 61 years of age. All subjects reported being native English speakers. A subgroup of 39 subjects (22 females) aged, on average, 33.6 (±12.7) years old took part in study 1. Their mean level of education was 15.1 (±2.3) years. Study 2 was carried out with a second subgroup of 33 subjects (17 females) with a mean age of 36.7 (±12.9) years old and a mean level of education of 14.5 (±2.7) years. Students composed respectively 35 and 27% of the subjects in the subgroups 1 and 2. According to the Research Ethic Board of the Douglas Institute, acquiring descriptive normative data from visual stimuli is a procedure that does not require ethical approval. The Research Ethic Board of the Douglas Institute thus waived the need for consent from our subjects. Prior to the normative session, subjects were nevertheless explained that they were free to interrupt their participation at any time and for any reason.

Although our sample of subjects was comparable to those used in many other normative datasets, the reliability of our measures scored on a scale of 1 to 5 was tested by splitting the data randomly in two subject groups. Spearman-Brown split-half reliability coefficients to all normative variables were over .8 (familiarity: .872, visual complexity: .912, object agreement: .896, viewpoint agreement: .847, and manipulability: .950), and thus very acceptable.

### Stimuli

**Figure 1:**
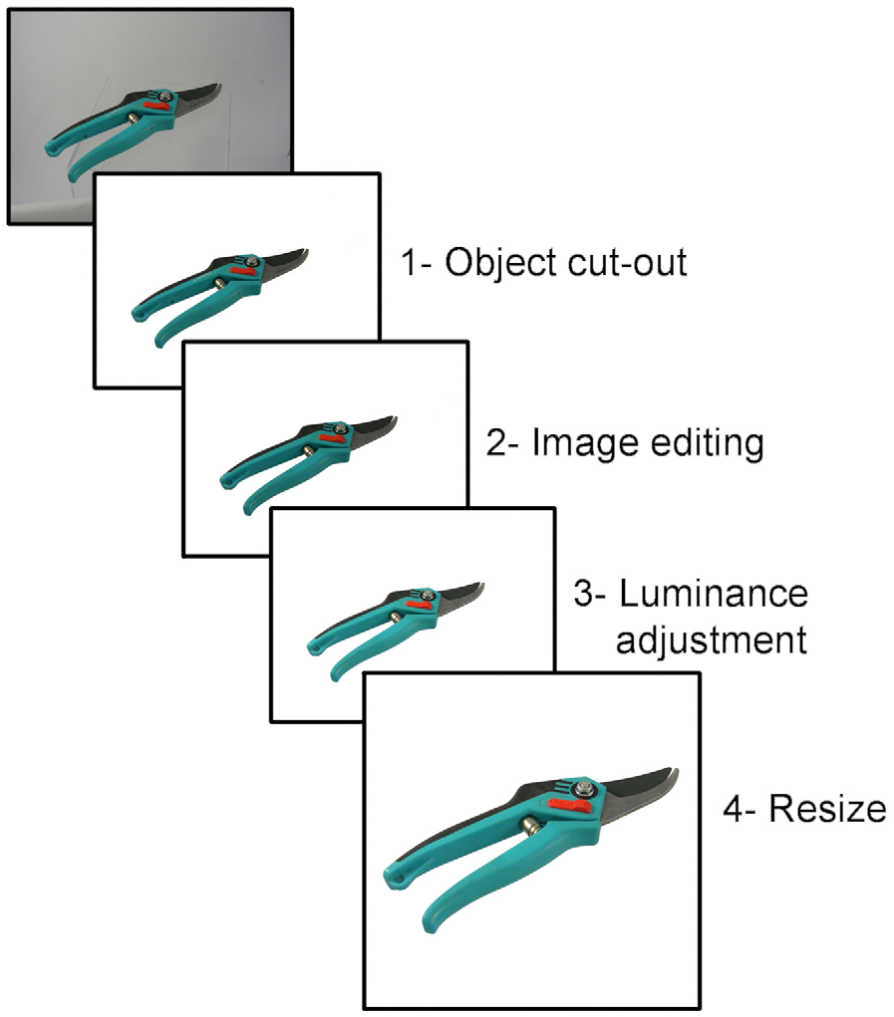
The 5-step procedure of stimulus creation.

**Figure 2:**
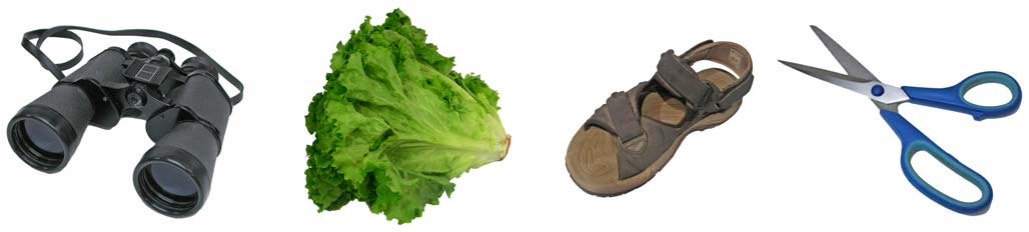
Some examples from the stimulus set.

A large set of 1,460 photo stimuli were created through a 5-step procedure, presented in Figure 1. First, common objects were gathered and digitally photographed one at a time at a pixel resolution of 2816×2112 (300 dpi). Most objects were photographed in a box that uniformly diffused the light provided by two projectors. Adobe Photoshop (Adobe Systems Inc., San Jose, U.S.A.) was used for image editing, which essentially consisted in 1) cutting-out the object from the scene by turning the background to white, 2) removing stains, brand names, company logos, and other prominent words, 3) adjusting the colors, the lightness and the contrast in a way that improved the visibility of the object, and 4) resizing the object and placing it in a frame of 2000×2000 pixels. Some functions of Photoshop and CorelDraw software (Corel Corp., Ottawa, Canada) were applied to attenuate the areas of shade, to equalize the luminosity and color within the images, and to accentuate the visibility of the contours where necessary. Some of the objects were new but many were old or used. In such cases, the objects were cleaned and the image was edited using Photoshop as necessary. Many of the photos included the same objects photographed from different viewpoints, or different exemplars of the same type of objects. Unless the exemplar is being used as an experimental condition, one would normally avoid using more than one exemplar in a set of stimuli because they bear the same name. For the normalization, we thus reserved only one exemplar per object or more specifically one object of all those sharing the same names in English or in French (for another study). This subset included 538 unique exemplars. Of the photo stimuli submitted to normalization, 58 were removed from the bank either because they were unrecognized by too many subjects and had a DKO score (see below) over 20% (e.g., 26% of the subjects could not recognize the shoe horn), named incorrectly (e.g., the apricot was named a peach by a majority of subjects), or because less than 20% of the subjects named the object similarly (e.g., the bag tie was given 11 different names and reached a name agreement of 19%) . The BOSS includes 480 photo stimuli. Some of these stimuli are presented in Figure 2.

**Figure 3:**
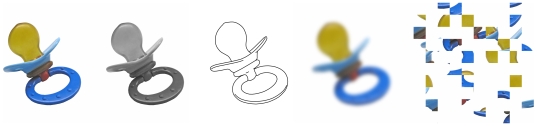
Example of one object presented in four different conditions. Note that objects are blurred and scrambled at 10 and 8 different levels respectively and the drawing version is currently available for 149 stimuli.

Some sets of visual stimuli come with altered versions built specifically to serve as control conditions. For instance, object processing of Snodgrass and Vanderwart's pictures was controlled by fragmenting line-drawn contours [67]. The surface quality of the photo stimuli makes such manipulation impossible. However, there are other ways to create ‘no object’ conditions. One way is to scramble pieces of the images (e.g., [68]). It is also possible to attenuate the contrast, add noise or to make the image blurry [65]. Because most of these manipulations are parametrically defined, the likeliness of recognizing the objects can be modulated. We here propose our set in four different altered versions, all presented through an example in Figure 3. The first is a grayscale version that can be used to control for color processing. Some stimuli, 137 to date, have also been reproduced in black-and-white line-drawn versions and can serve to control for pictorial format. It is important to remember, however, that the norms of the present study are applicable neither to the grayscale nor the line-drawn versions. For the scrambled versions, the images were broken down into square tiles rearranged randomly, like a sliding block puzzle. The size of the square tiles was adjusted to either 50, 100, 150, 200, 250, 300, 350, or 400 pixels in width and height in order to generate eight degrees of scrambled conditions. Finally, ten blurred versions were created by applying a Gaussian filter with a radius starting at 10 pixels and increasing up to 100 by adding 10 pixels per level. All of these versions are available along with the original versions of the photo stimuli.

### General procedure

Random sequences of all photo stimuli were generated. The sequences were managed with E-Prime software (Psychological Software Tools, Pittsburgh, PA) by a laptop connected to an Optoma EP7150 DLP projector (1024×768 XGA). The calibration of the RGB colors display was verified using the calibratemonspd function of psychtoolbox and a Spectrascan 650 colorimeter.

Subgroups of 7 to 15 subjects were brought to a conference room and handed response sheets on which they first had to indicate their age, gender, and years of education. The sheets included numbered lines, one for each object, on which they noted their responses. All objects were presented one at a time every 20 seconds. The pace was established beforehand to provide subjects with sufficient time to write their responses. The order of the stimulus sequence was different across groups in order to avoid order sequence effects.

### Study 1

The goal of the study 1 was to normalize the photo stimuli regarding three of the variables defined by Snodgrass and Vanderwart [5], namely name, familiarity and visual complexity. Category was added as a fourth variable. Categories of stimuli are usually decided by scientists who conduct a study, although they are subject to inter-individual differences. Normalizing across the category variable provides a measure of such differences by indicating how subjects agree to include an object within the same category. With such an agreement score, it will be possible to identify the most representative objects of their category. In addition to the category norms, we classified the objects as living and non-living. This classification essentially distinguished organic objects from inanimate and man-made objects and was not submitted to normalization.

In Study 1, instructions were given orally but they were also described on a sheet handed to each subject (see Table 1). For the name, subjects were asked to: “Identify the object as briefly and unambiguously as possible by writing only one name, the first name that comes to mind.” It was specified that the name could be composed of more than one word. As in Snodgrass and Vanderwart [5], they were instructed to write DKO (don't know object) if they had no idea what the object was. If they knew the object but not the name, they wrote DKN (don't know name) and if they knew the name but were unable in the moment to retrieve it, the instructions were to write TOT (tip-of-the-tongue). For the category, subjects had to make a selection across 18 categories and an “others” choice. Categories included building materials, clothing, decoration and gift accessories, electronic devices and accessories, food, furniture, games, toys and entertainment, hand labour tools and accessories, household articles and cleaners, jewels and money, kitchen utensils, medical instruments and accessories, musical instruments, natural elements and vegetation, outdoor activity and sport items, skin care and bathroom items, stationary and school supplies, and weapons and items related to war. These categories were sorted in alphabetical order on a sheet. The instruction was to: “Determine in which category the object belongs to.” The subjects had to write the number assigned to the category they chose. It was clearly stated that “others” should be used only if no proposed category satisfied their own criteria and they were discouraged to use this option if their intention was to be more specific regarding the category. For familiarity, subjects were asked to: “Rate the level to which you are familiar with the object.” Their response was provided on a 5-point rating scale with 1 indicating very unfamiliar and 5, very familiar. Subjects were provided with clear instructions that they had to rate the concept itself rather than the picture of the object. They were also instructed and encouraged to use the full value of the scale. Responses were not required when they answered DKO for the name. Finally, for visual complexity, subjects were asked to “Subjectively rate the level to which the image appears to be complex in terms of the quantity of details and the intricacy of the lines.” Value 1 indicated a very simple image and 5, a very complex image.

**Table 1:** Instructions.

Study 1 (n = 39)	Instruction
Name	Identify the object as briefly and unambiguously as possible by writing only one name, the first name that comes to mind
Category	Determine in which category the object belongs to
Familiarity	Rate the level to which you are familiar with the object
Visual complexity	Subjectively rate the level to which the image appears to be complex in terms of the quantity of details and the intricacy of the lines

Study 2 (n = 33)	
Object agreement	How closely the picture resembles the mental image you had for the object name, independently from its position?
Viewpoint agreement	How closely the object is positioned as the object you imagined?
Manipulability	Could you easily mime the action usually associated with this object so that any person looking at you doing this action could decide which object goes with this action?

### Study 2

Image agreement is the fourth norm that usually accompanies name, familiarity and visual complexity. Image agreement is the degree to which the mental image generated out of the modal name matches the object stimulus. In the original instruction for this norm [5], there was no specific criterion for deciding how well images matched. Image agreement could thus be based on a matching in terms of the objects' design but also on how well positions were matched. In the present study, object design (i.e., structure) and viewpoint were tested separately. Subjects thus had to decide to what extent the mentally generated object was structurally similar to the photo object and to what extent the two objects had comparable positions. Such norms could be indicative of how typical the object and its viewpoint are. Surprisingly, typicality has rarely been tested directly [10], [12]. It nevertheless represents a well studied variable affecting memory recall and recognition of objects. For instance, it is easier to recall objects that have been encoded while presented in the most familiar viewpoint [72] or to recognize objects presented from a familiar viewpoint [73]. By normalizing viewpoint agreement, it will become possible to determine the extent to which the object is displayed from a typical viewpoint and, therefore, to control for the potential effects this variable might have on cognitive performances such as recall and recognition. Manipulability was added as a third variable to be normalized in this study. As explained in the introduction, manipulability is a variable that influence behavioral performance on cognitive tasks [50] and thus deserves to be normalized.

In Study 2, each presentation started with the appearance of a word displayed on the screen for five seconds. The word was the modal name of the object. The name was immediately followed by the appearance of its corresponding photo stimulus and remained on the screen for 15 seconds. The instruction for the object agreement norm was to judge: “How closely the picture resembles the mental image you had for the object name, independently from its position.” Prior to the testing session, subjects were told that each time a word appeared, they were allocated five seconds to imagine the object depicting this word. When the picture appeared, they had to determine on a 5-point scale to what extent the actual object corresponded to the mental image they had generated. A value of 1 signified low agreement and a value of 5 signified high agreement. For the second norm, viewpoint agreement, the subjects were instructed to determine: “How closely the object is positioned as the object you imagined.” Again, they responded on a 5-point scale where 1 signified low agreement and 5 signified high agreement. Subjects were told that their rating should not take into account the difference of orientation or reflection between the objects. The profile of a car pointing toward the right and another profile pointing toward the left, for instance, are perceived from the same angle of view and should not deserve a low score on the scale. An example was provided to help subjects understand the task: The word airplane was presented followed by a photo of a Boeing airliner, which was described as the image one could potentially have imagined for the word airplane. We then presented the photo of another Boeing airliner positioned from a different viewpoint and explained that both were very similar and thus deserved a high score on object agreement but a low score on viewpoint agreement. We repeated the procedure with a Cessna positioned like the Boeing airliner as the imagined airplane. This served as an example that deserved a low score on object agreement but a high score on viewpoint agreement. Subjects were instructed to write NMI (no mental image) beside the scale when they were unable to generate a mental image or when they did not know to what object the name was referring to. For manipulability, we used the instruction described by Magnié, Besson, Poncet, and Dolisi [22] which consisted in asking: “Could you easily mime the action usually associated with this object so that any person looking at you doing this action could decide which object goes with this action?”. Again, responses were provided on a 5-point scale. A value of 1 was assigned to a definite “no” response and a value of 5 was assigned to a definite “yes” response.

### Analyses

#### Modal name

For each object, the percentage of subjects using each name was computed after the exclusion of the DKN, DKO, and TOT responses. The name reaching the highest percentage was identified as the modal name. The percentage corresponded to the modal name agreement. When two names reached the same percentage, the most precise name (e.g., plastic cup as opposed to cup) was preferred. Adjectives used to describe a state (e.g., empty glass) or a feature that was totally irrelevant for the identity of the object (e.g., white candle) were discarded. Adjectives counted as long as they provided relevant information regarding the nature, the shape, or the function of the object. For instance, the adjective in “girl sock” is highly relevant since it helps define a specific type of sock. In fact, adjectives were discarded only in rare instances. It should also be noted that composite names with the same words placed in different order (e.g., bottle of oil and oil bottle) were compiled as the same name.

#### H value

The statistic *H* is a value sensitive to the number and weight of alternative names. It is computed with the following formula:
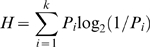
where *k* refers to the number of different names given to each picture and excludes the DKN, DKO, and TOT responses. *P_i_* is the proportion of subjects who gave a name for each object. It should be noted that this proportion varies across objects because of the exclusion of the DKN, DKO, and TOT responses. The *H* value of an object with a unique name and no alternative will be 0. The *H* value of an object with two names provided with an equivalent frequency will be 1.00. This value will be smaller for an alternative that is provided to a lower frequency rate. On the other hand, the *H* value will increase as a function of the number of alternatives. For instance, one modal name of 50% frequency and two alternatives of 25% frequency each will give an *H* value of 1.50.

#### Category agreement and H_cat_ value

A modal category and an *H* value, referred to here as an *H*_cat_ value, were computed following the same procedure used for the names. Unlike for the name, objects could have more than one category.

#### Variables rated on a 5-point scale

Familiarity, visual complexity, object agreement, viewpoint agreement, and manipulability were computed by averaging the scores on the 5-point rating scale.

## Results and Discussion

**Figure 4:**
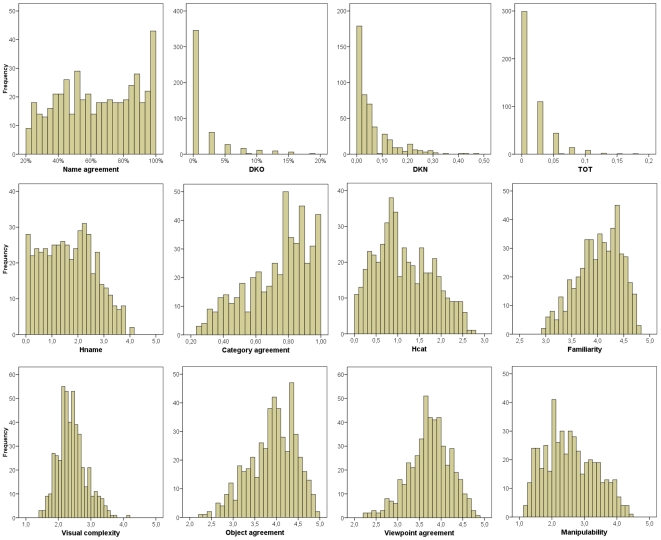
Graphical display of tabular frequencies of norms.

Table 2 summarizes the norms of the 480 photo of the BOSS and Figure 4 depicts their histograms. Norms are presented in Table 3 as a function of the categories. These norms are averages. To consult stimulus-specific norms, please refer to Appendix S1. In this Appendix, photo stimuli are sorted as a function of their filename. The filename was preferred to the modal name because this latter was sometimes not specific enough.

**Table 2:** Norms.

Variables	Mean	SD	Min	Max
Modal name agreement	64%	23%	20%	100%
*H* value	1,65	1,01	0,00	4,10
DKO	2%	3%	0%	18%
DKN	6%	8%	0%	47%
TOT	2%	3%	0%	18%
Category agreement	73%	19%	26%	100%
*H*_cat_ value	1,15	0,65	0,00	2,70
Familiarity	4,0	0,4	3,0	4,8
Visual complexity	2,4	0,4	1,4	4,1
Object agreement	3,9	0,5	2,3	4,9
Viewpoint agreement	3,7	0,5	2,2	4,8
NMI	2%	4%	0%	30%
Manipulability	2,6	0,8	1,2	4,5

DKO = Don't know object; DKN = Don't know name; TOT = Tip-of-the-tongue.

NMI = No mental image.

**Table 3:** Norms as a function of categories.

Category	Nb	NA	*H*	DKO	DKN	TOT	CA	Hcat	Fam	VC	OA	VA	Man
Building materials	3	75%	1,13	3%	6%	1%	58%	1,69	3,7	2,1	3,9	3,8	1,7
Clothing	28	69%	1,43	0%	2%	1%	81%	0,80	4,3	2,3	3,6	3,5	3,0
Decoration and gift accessories	27	58%	1,93	2%	8%	1%	62%	1,57	3,7	2,7	3,4	3,5	2,2
Electronic devices and accessories	36	56%	2,03	1%	3%	1%	78%	1,06	4,1	2,9	3,9	3,7	2,6
Food	78	76%	1,13	2%	3%	1%	81%	0,78	4,3	2,3	4,3	4,0	1,8
Furniture	2	72%	1,20	0%	0%	0%	51%	1,87	4,5	2,5	3,3	4,0	3,0
Games, toys and entertainment	23	54%	2,04	3%	6%	2%	77%	0,99	3,7	2,5	3,7	3,7	2,5
Hand labour tools and accessories	37	62%	1,81	2%	16%	3%	72%	1,20	3,7	2,4	3,9	3,7	2,8
Household articles and cleaners	29	61%	1,84	2%	6%	2%	57%	1,71	4,0	2,3	3,9	3,6	2,7
Jewels and money	8	73%	1,28	2%	3%	1%	61%	1,67	4,2	2,5	3,6	3,6	3,1
Kitchen utensils	60	58%	1,93	2%	9%	2%	82%	0,87	4,0	2,3	3,8	3,8	2,6
Medical instruments and accessories	9	64%	1,71	4%	7%	3%	63%	1,57	3,7	2,5	4,0	3,8	3,1
Musical instruments	4	75%	1,31	4%	12%	5%	78%	1,20	3,6	3,0	4,1	4,3	3,5
Natural elements and vegetation	11	62%	1,61	2%	3%	1%	69%	1,29	3,9	2,6	3,8	3,8	2,0
Outdoor activity and sport items	18	60%	1,84	2%	4%	2%	72%	1,21	3,9	2,5	4,1	3,9	2,9
Skin care and bathroom items	32	63%	1,68	1%	5%	1%	73%	1,19	4,1	2,3	3,9	3,6	3,3
Stationery and school supplies	38	67%	1,44	1%	5%	2%	85%	0,75	4,2	2,2	4,0	3,6	2,6
Weapons and items related to war	0	-	-	-	-	-	-	-	-	-	-	-	-
Others	36	65%	1,66	1%	4%	2%	48%	2,00	3,9	2,4	3,9	3,5	2,7

NA = Modal Name Agreement; *H* = *H* value; DKO = Don't know object; DKN = Don't know name; TOT = Tip-of-the-tongue; CA = Category Agreement; *H*cat = *H* value for category; Fam = Familiarity; VC = Visual Complexity; OA = Object Agreement; VA = Viewpoint Agreement; Man = Manipulability.

### Names

A comparison with normative datasets of line-drawn pictures shows that the modal name agreement is low and the *H* value is high. Modal name agreement reached 64% (±23%) as compared to agreement, which ranged between 72% (±23%) and 85% (±16%) (depending on language) reported in other studies using line-drawn pictures [17]. Such a result thus indicates that the name most frequently reported to identify an object in the present study was on average used by fewer subjects than in other studies. With a mean of 1.65 (±1.01), the *H* value was numerically higher in the present study than in the studies of Snodgrass and Vanderwart [5] and Bates and colleagues [17] which reported *H* values of .56 (±.53) and .67 (±.61) to 1.16 (±.79). Such a finding indicates that the present subjects used more alternative names to identify the objects.

Differences of object selection between the BOSS and the other normative datasets are largely responsible for the difference of modal name agreement and *H* value. We carefully examined the Snodgrass and Vanderwart set and found 97 drawings depicting objects that could also be found in the present set. These objects are identified in Appendix S1 by an asterisk symbol. The modal name agreement and *H* value for these 97 objects were respectively of 87.9% (±13.5) and 0.50 (±0.49) in Snodgrass and Vanderwart (1980) and of 82.9% (±17.2) and 0.82 (±0.74) in the present study. These statistics still show higher agreement for the line-drawn pictures but the difference is now fairly small. The modal name agreement of the BOSS was thus likely reduced, and the *H* value, mainly increased, because of the other objects found only in our set. It is important to consider that Snodgrass and Vanderwart (1980) selected objects that were typical and that would likely lead to high modal name agreement. To gather as many objects as we did for the BOSS, we could not fulfill this condition and we had to include objects that were likely difficult to name appropriately. The 480 objects included in the BOSS are nevertheless all recognizable as indicated by a DKO rate of only 2% (±3%). The fact that objects are recognizable does not necessarily means that they are easily named. The sum of DKN (6%±8) and TOT (2%±3) was relatively high. Having more objects thus comes at the expenses of a reduction of modal name agreement and an increase of the *H* value. This expense was also observed with line-drawn pictures. For instance, Cycowicz and colleagues (1999) reported a name agreement of 67.44% for an additional set of 61 pictures (set 2), and 73.18% for another additional set of 79 pictures (set 3). The name agreement for these two sets was far below the 86.65% obtained for the 260 Snodgrass & Vanderwart original pictures. As another example, Bonin and colleagues (2003) have created a set of 299 new pictures to complement the Snodgrass and Vanderwart set. They obtained a modal name agreement of 77.4% and an *H* value of 0.67. These statistics were respectively lower and higher than those reported by Snodgrass and Vanderwart (1980). These differences were not as large as those observed with the present norms but it must be kept in mind that our set of photo stimuli faced the constraint of including only common objects.

Other reasons can be put forward to explain why name agreement for the 97 objects common to our set and to the set of Snodgrass and Vanderwart (1980) were slightly lower in our set. First, photo features such as color can sometimes be helpful but can also interfere with the task. For instance, color helped reach a modal name agreement of 100% for the orange in the present study. This agreement was only of 81% for the black-and-white drawing version (Snodgrass & Vanderwart, 1980). In contrast, the modal name of the pepper in the present study was of 45%, lower than the 67% reported by Snodgrass and Vanderwart (1980). The difference was essentially because subjects wrote either “pepper” or “red pepper.” The details found in photos could also lead to a similar balance of advantages and disadvantages. On the one hand, details can be useful to better identify the objects, but on the other hand, they can bring the subjects to give a more precise response that takes into account some physically and functionally idiosyncratic features of the objects. For instance, the box in the present set has a decorative design, and was consequently named “gift box” and “decorative box” by 26% and 10% of the subjects, respectively. Since no such details were found in the line-drawn box of Snodgrass and Vanderwart (1980), subjects were prone to simply name it “box.” Evolution of the consumer products is another factor that may likely account for the lower modal name agreement in the present study. The development of recent technologies has led to a diversification of products. Today, a telephone can have many forms (e.g., touchtone phone, cell phone, e-phone, wireless phone, etc.) and this necessarily requires names to be more specific.

### Categories

The category with the greatest number of objects was food (78) and kitchen utensils (60). The category of hand labour tools and accessories, which is widely used, included 37 objects. Norms collected throughout the two studies are presented in Table 3 as a function of each category. Note that the number of objects included in some categories was very low. The category reaching the highest agreement was stationary and school supplies, followed by kitchen utensils, clothes, and food. Agreement for food items was however lowered by subjects classifying these items in the natural elements and vegetation. Food and hand labour tools were more closely examined as they are frequently compared in studies on categorization processing. Results indicated that modal name agreement and category agreement were both higher for the food category. Food items were also more familiar on average but they did not differ from tools regarding visual complexity. Tools, conversely, reached a higher manipulability score. The objects were also classified depending on whether they were living or non-living things. Overall, there were fewer living objects (60) than non-living objects (420). Most living objects, 54, were food items. The remaining living objects were five natural elements and vegetation and one decoration item.

### Familiarity and visual complexity

The familiarity and the visual complexity average ratings ranged over a scale from 1 to 5 (5 being very familiar and very complex), and they were respectively of 4.0 (±.4) and 2.4 (±.4). These values are numerically higher than the familiarity score of 3.3 (±1.0) but lower than the visual complexity score of 3.0 (±.9) reported by Snodgrass and Vanderwart [5]. Higher familiarity is not surprising given that most of the present objects were daily-used objects. On the other hand, one could have expected a higher visual complexity score for the present photos as such stimuli include more details than drawings. However, photo stimuli are more similar to what subjects are used to perceiving everyday. The texture of a towel, for example, should not appear as something particularly complex. In a drawing, texture can look artificial, lead to some ambiguities and create an impression of visual complexity. Moreover, lines in drawings are much more contrasted than edges in photo stimuli. This could increase the impression of complexity, particularly in line-drawn pictures with many lines (e.g., the train).

### Object agreement, viewpoint agreement, and manipulability

The average object and viewpoint agreements between a mental image and the photo stimulus reached respectively 3.9 (±.5) and 3.7 (±.5) on the 5-point scale (with highest value indicating full agreement). These results are consistent with the image agreement of 3.4 to 3.8 generally reported for pictures [5], [9], [33], [39], [46]. A low object agreement could have been expected considering that objects had particular designs. The high rate of agreement thus suggests that in general, the objects of the BOSS are typical and presented from a standard viewpoint. The mean rate for manipulability was 2.6 (±.8), which is smaller than the rate reported by Magnié and colleagues [22] who found that weakly manipulable objects had a rating of 3.3. A greater homogeneity in terms of manipulability in the present study might have lead subjects not to use the full range of rating values and to generally use the middle value. As for the NMI (no mental image) it should be noted that such variable is applicable to the name and not to the photo stimulus. It can be associated in some ways to the imageability, that is the propensity of a word to evoke various images [33].

### Correlations

In each normative study, description of the norms is generally followed by correlational analyses that examine how each norm is related to the other norms. The correlations reported in 15 studies [5], [7], [9], [10], [11], [12], [29], [33], [34], [36], [39], [44], [46], [69], [70] can be summarized as follows: First, in each of the 15 studies, modal name agreement and the *H* value are negatively correlated. This correlation is above .900 in seven out of 11 studies. The second most consistent finding is a negative correlation between familiarity and visual complexity which is usually around .400. Positive correlation between modal name agreement and familiarity and negative correlation between modal name agreement and visual complexity have sometimes been found but they were rarely very significant. In fact, in half of the studies testing these correlations, results were not significant [5], [7], [34], [36], [39], [46].

**Figure 5:**
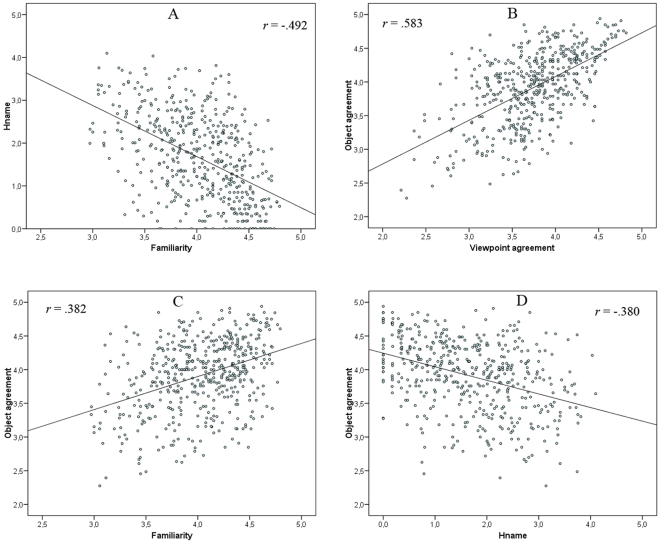
Scatter plots of the correlations. Correlations are between A) H value and familiarity, B) object and viewpoint agreement, C) object agreement and familiarity, and D) object agreement and H value.

Pearson correlations between norms of the present photo stimuli have been examined, and are presented in Table 4. The .05 significance level was Bonferroni corrected. The scatter plots of the most relevant correlations are also presented in Figure 5. As is usually found with line-drawn pictures, modal name agreement and the *H* value were the most strongly correlated variables (−.960 with confidence intervals, CI, of .952 and .966). Modal name agreement and the *H* value were also both strongly correlated with familiarity (.421, CI: .344 and .492; and −.492, CI: −.557 and −.421) but not with visual complexity (−.108 and .109). The correlation between the *H* value and familiarity is the highest ever reported and thus contrasts with the inconsistency of such correlation across studies using line-drawn pictures. The present correlation is not surprising as it has been shown that familiar stimuli are named more easily than unfamiliar stimuli [71]. On the other hand, there was no correlation between the norms related to the name and visual complexity. Such a result parallels the weakness of this correlation in normative datasets of line-drawn pictures. The most intriguing result is the absence of a significant correlation between familiarity and visual complexity that is routinely observed in normative datasets of line-drawn pictures. Ten out of eleven studies reported a significant correlation [5], [7], [9], [29], [33], [34], [36], [39], [46], [70]. Familiarity might be responsible for this absence of significance. Most of the present objects were indeed familiar, so a low score on the familiarity scale might not be equivalent to the same rating performed with the line-drawn pictures. A greater variability of familiarity might thus have been needed for the correlation between visual complexity and familiarity to be significant. Overall, except for the lack of correlation between familiarity and visual complexity, the correlational patterns in the present data are very similar to those observed in normative datasets of line-drawn pictures.

**Table 4:** Matrix of correlations.

Study		Modal name agreement	*H* value	Familiarity	Visual complexity	Category agreement	*H*_cat_ value	Object agreement	Viewpoint agreement
1	*H* value	−0.960*							
	Familiarity	0.421*	−0.492*						
	Visual complexity	−0.108	0.109	−0.154					
	Category agreement	0.068	−0.083	0.296*	−0.077				
	*H*_cat_ value	−0.106	0.126	−0.348*	0.093	−0.954*			
2	Object agreement	0.326*	−0.380*	0.382*	−0.089	0.213*	−0.255*		
	Viewpoint agreement	0.178*	−0.191*	0.210*	−0.0401	0.158	−0.174	0.583*	
	Manipulability	0.237*	−0.261*	0.197*	0.070	−0.014	0.054	0.078	−0.040

Correlations significant to the .05 level, Bonferroni corrected, are marked with an asterisk.

The most important correlation involving variables of study 2 was of .583 (CI: .521 and .639), between object and viewpoint agreement, and was followed by a correlation of .382 (CI: .303 and .456) between object agreement and familiarity. Both correlations were statistically significant. Considering the nature of these two variables, such correlations were to be expected. They do, however, contrast with the results observed in normative datasets of line-drawn pictures where image agreement and familiarity are generally uncorrelated. For instance, only two studies out of eight reported a significant correlation but both were very low and in opposite directions (.138 in Snodgrass & Vanderwart, 1980 and −.155 in Sanfeliu & Fernandez, 1996). Correlations in the present study might have been bolstered by the fact that most objects were familiar. In the Snodgrass and Vanderwart set, some objects were not very familiar (e.g., a lion) but reached a high level of image agreement. Object agreement was also significantly correlated with the *H* value (−.380, CI: −.454 and −.301) and with modal name agreement (.326, CI: .244 and .404) but not with visual complexity. Correlation with name feature is unsurprising as subjects were presented with the modal name to generate their mental image. The fact that the correlation was higher in the present study than in those with line-drawn pictures is thus simply due to lower name agreement in the present study. It is also consistent with the fact that more typical objects are named faster [74]. Viewpoint agreement presented the same correlational pattern but to a smaller extent. Viewpoint and object agreement may thus both contribute to what was referred to as image agreement in previous studies, but their respective contribution nevertheless differs. Finally, manipulability correlated weakly but significantly with only a few other variables including modal name agreement (.237, CI: .151 and .320), *H* value (−.261, CI: −.343 and −.176), and familiarity (.197, CI: .109 and .282). Magnié and colleagues [22] reported a strong correlation between manipulability and familiarity but this result was much weaker after they focused their analyses on manipulable objects only. Contrary to what could have been expected; manipulability did not correlate with viewpoint agreement, thus suggesting that these variables are independent.

Norms collected in each study were all provided using the same sample of subjects and thus subject to possible synergistic influences across the different dimensions being rated. For instance, subjects might be prone to rate an object with a lower familiarity score if they had difficulty elaborating a name for the object. This might be seen as an explanation for the very high correlation between familiarity and norms related to the names. Of the eleven studies with drawing pictures testing the correlations between modal name agreement, familiarity and visual complexity, five collected the norms from the same subjects [6], [7], [34], [36], [46]. Surprisingly, no significant correlation was found between modal name agreement and familiarity, and between modal name agreement and visual complexity, except in the study by Cycowicz and colleagues [6, set 3], where modal name agreement and familiarity significantly correlated. However, this correlation only included 79 drawing pictures. In the six studies testing each variable by a different subgroup of subjects, modal name agreement was sometimes correlated with familiarity [9], [11], [29], [33] and sometimes with visual complexity [5], [9], [29]. In light of these results, using the same or different subjects to normalize variables likely influences the correlation of the resulting norms but it cannot at this point be easily determined which methods should have been privileged. On the one hand, using the same subjects increases the likelihood that one variable's rating influences the rating of another variable. On the other hand, this problem is avoided by testing each variable with different subjects, though in such a condition, correlations are likely smaller because they are calculated between subgroups.

### General discussion

The current project proposes a large set of ecological stimuli for research in cognition, vision, and psycholinguistics. There are other normative datasets available but the present one, the BOSS, offers photo stimuli of high quality, all collected in identical conditions. Moreover, the BOSS proposes the greatest number of photo stimuli, 480, that have ever been normalized. The classic norms have been collected as well as new ones providing an indication on the manipulability of the objects, the category to which they belong, and the extent to which their position and design are typical.

Each of the normative variables contributes to a better definition of the stimuli. Beyond their descriptive value, normative variables are interesting in that they can influence various kinds of cognitive processing and generate unique brain activities. For instance, objects of different categories activate selective patterns of the brain within the dorsal occipital cortex, the superior temporal sulcus, and the ventral temporal cortex [75], [76]. Chao and Martin [77] showed that viewing and naming pictures of tools activates a neural network within the ventral premotor and the posterior parietal areas that is not activated by non-manipulable objects. Change of viewpoint can alter neural activity in the ventral temporo-occipital cortex (area vTO) [78]. It has also be shown that activity in the dorsolateral prefrontal region is influenced by whether the view of an object is atypical (non-canonical) [79]. When they are not used as an the experimental variable, the normative variables should thus be imperatively controlled in order to avoid any undesired and confounding influences they might otherwise exert on performance and, if the experiment involves a brain imaging technique, on brain activities (see [71]). This controlling procedure is systematically applied in psycholinguistics research and should be used in research using pictures. When they define the conditions for an experiment, scientists should thus ensure that stimuli have comparable modal name agreement, familiarity, visual complexity, etc. across conditions. These control measures are of capital importance in light of the growing body of evidence showing that the influence of one variable relies on the presence of another variable. For instance, Filliter and colleagues [50] showed that manipulable objects were identified faster than non-manipulable objects. When familiarity was controlled, however, the difference was reversed, with the non-manipulable objects now being identified more quickly.

Sets of line drawings and sets of photos of objects are complementary tools suitable for different experimental requirements. Line drawings are schematic and simplified representations in which only the most relevant features are depicted. As explained by Snodgrass and Vanderwart (1980), these pictures are made to be the most typical and unambiguous representations of a concept. Therefore, scientists interested specifically in the processing of concepts as opposed to the visual stimuli themselves will probably find the Snodgrass and Vanderwart set more suitable for their experimentation. On the other hand, the BOSS offers norms for photo stimuli. Because it includes only commonly used objects, the BOSS cannot satisfy the needs of research using categories of stimuli such as animals, buildings and vehicles. Other sets, such as the set of Viggiano and colleagues [65], offer such stimuli. However, the number of photo stimuli in the BOSS is substantially higher than in other sets of normative photos. This is a valuable feature for scientists who need a large number of stimuli across their experimental conditions. As we explained in the results and discussion section of study 1, the number of stimuli was obtained at the expense of lower modal name agreement and higher *H* value. Scientists interested in using only stimuli with high modal name agreement still have the option of taking only those with high modal name agreement in the 480 photo stimuli set. There are, for instance, 211 photo stimuli with a modal name agreement of 70% or more (for a mean of 87%). However, having a wide range of values across one norm, such as modal name agreement, can be of particular interest. As Adlington and colleagues [66] argued, having a wide range of values facilitates the measure of a naming effect and prevents potential ceiling effects.

There will always be a need for normative stimuli of high quality. The Snodgrass and Vanderwart set [80] has been one of the most important resources of visual stimuli used in vision, cognitive and psycholinguistics research. In the last decade, the need for photo stimuli has grown, and we hope that the present set, in combination with the other existing sets of photo stimuli, will contribute in fulfilling this need. Norms from the BOSS apply to subject samples of Canadians and, to an extent, North Americans. Because of cultural factors, however, they should be used with discretion in other countries. Further expansion of the BOSS should thus start with the collection of norms across different countries and different languages, as with the Snodgrass and Vanderwart set.

## Supporting Information

Appendix S1 Normative Data of the 480 photo stimuli.
(0.19 MB XLS)

## References

[1] Coltheart M (1981). The MRC Psycholinguistic Database.. The Quarterly Journal of Experimental Psychology.

[2] Gilhooly KL, Logie RH (1980). Age of Acquisition, imagery, concreteness, familiarity and ambiguity measures for 1944 words.. Behavior Research Methods, Instruments, & Computers.

[3] de Groot AMD (1989). Representational aspects of word imageability and word frequency assessed through word association.. Journal of Experimental Psychology: Learning, Memory, & Cognition.

[4] Schiano DJ, Watkins MJ (1981). Speech-like coding of pictures in short-term memory.. Memory & Cognition.

[5] Snodgrass JG, Vanderwart M (1980). A standardized set of 260 pictures: norms for name agreement, image agreement, familiarity, and visual complexity.. Journal of Experimental Psychology: Human Learning & Memory.

[6] Cycowicz YM, Friedman D, Rothstein M, Snodgrass JG (1997). Picture naming by young children: norms for name agreement, familiarity, and visual complexity.. Journal of Experimental Child Psychology.

[7] Berman S, Friedman D, Hamberger M, Snodgrass JG (1989). Developmental picture norms: Relationships between name agreement, familiarity, and visual complexity for child and adult ratings of two sets of line drawings.. Behavior Research Methods, Instruments, & Computers.

[8] Dunn L, Dunn L (1981). Peabody picture vocabulary test-revised:.

[9] Bonin P, Peereman R, Malardier N, Meot A, Chalard M (2003). A new set of 299 pictures for psycholinguistic studies: French norms for name agreement, image agreement, conceptual familiarity, visual complexity, image variability, age of acquisition, and naming latencies.. Behavior Research Methods, Instruments, & Computers.

[10] Alvarez B, Cuetos F (2007). Objective age of acquisition norms for a set of 328 words in Spanish.. Behavior Research Methods.

[11] Nishimoto T, Miyawaki K, Ueda T, Une Y, Takahashi M (2005). Japanese normative set of 359 pictures.. Behavior Research Methods.

[12] Dell'Acqua R, Lotto L, Job R (2000). Naming times and standardized norms for the Italian PD/DPSS set of 266 pictures: direct comparisons with American, English, French, and Spanish published databases.. Behavior Research Methods, Instruments, & Computers.

[13] Kremin H, Akhutina T, Basso A, Davidoff J, De Wilde M (2003). A cross-linguistic data bank for oral picture naming in Dutch, English, German, French, Italian, Russian, Spanish, and Swedish (PEDOI).. Brain & Cognition.

[14] Masterson J, Druks J (1998). Description of a set of 164 nounsand 102 verbs matched for printed word frequency, familiarityand age-of-acquisition.. Journal of Neurolinguistics.

[15] Schwitter V, Boyer B, Meot A, Bonin P, Laganaro M (2004). French normative data and naming times for action pictures.. Behavior Research Methods, Instruments, & Computers.

[16] Abbate MS, LaChapelle NB, Builders ACS (1984). Pictures, please! A language supplement..

[17] Bates E, D'Amico S, Jacobsen T, Szekely A, Andonova E (2003). Timed picture naming in seven languages.. Psychonomic Bulletin & Review.

[18] Himmanen SA, Gentles K, Sailor K (2003). Rated familiarity, visual complexity, and image agreement and their relation to naming difficulty for items from the Boston naming test.. Journal of Clinical and Experimental Neuropsychology.

[19] Szekely A, D'Amico S, Devescovi A, Federmeier K, Herron D (2003). Timed picture naming: extended norms and validation against previous studies.. Behavior Research Methods, Instruments, & Computers.

[20] Rossion B, Pourtois G (2004). Revisiting Snodgrass and Vanderwart's object pictorial set: the role of surface detail in basic-level object recognition.. Perception.

[21] Barbarotto R, Laiacona M, Macchi V, Capitani E (2002). Picture reality decision, semantic categories and gender. A new set of pictures, with norms and an experimental study.. Neuropsychologia.

[22] Magnie MN, Besson M, Poncet M, Dolisi C (2003). The Snodgrass and Vanderwart set revisited: norms for object manipulability and for pictorial ambiguity of objects, chimeric objects, and nonobjects.. Journal of Clinical and Experimental Neuropsychology.

[23] Boutsen L, Lamberts K, Verfaillie K (1998). Recognition times of different views of 56 depth-rotated objects: a note concerning Verfaillie and Boutsen (1995).. Percept Psychophys.

[24] Verfaillie K, Boutsen L (1995). A corpus of 714 full-color images of depth-rotated objects.. Percept Psychophys.

[25] Op de Beeck H, Wagemans J (2001). Visual object categorisation at distinct levels of abstraction: a new stimulus set.. Perception.

[26] Wagemans J, De Winter J, Op de Beeck H, Ploeger A, Beckers T (2008). Identification of everyday objects on the basis of silhouette and outline versions.. Perception.

[27] Panis S, De Winter J, Vandekerckhove J, Wagemans J (2008). Identification of everyday objects on the basis of fragmented outline versions.. Perception.

[28] De Winter J, Wagemans J (2004). Contour-based object identification and segmentation: stimuli, norms and data, and software tools.. Behav Res Methods Instrum Comput.

[29] Pompeia S, Miranda MC, Bueno OF (2001). A set of 400 pictures standardised for Portuguese: norms for name agreement, familiarity and visual complexity for children and adults.. Arquivos de Neuro-Psiquiatria.

[30] D'Amico S, Devescovi A, Bates E (2001). Picture naming and lexical access in Italian children and adults.. Journal of Cognition & Development.

[31] Cannard C, Blaye A, Scheuner N, Bonthoux F (2005). Picture naming in 3- to 8-year-old French children: methodological considerations for name agreement.. Behavior Research Methods.

[32] Masterson J, Druks J, Gallienne D (2008). Object and action picture naming in three- and five-year-old children.. Journal of Child Language.

[33] Alario FX, Ferrand L (1999). A set of 400 pictures standardized for French: norms for name agreement, image agreement, familiarity, visual complexity, image variability, and age of acquisition.. Behavior Research Methods, Instruments, & Computers.

[34] Weekes BS, Shu H, Hao M, Liu Y, Tan LH (2007). Predictors of timed picture naming in Chinese.. Behavior Research Methods.

[35] Yoon C, Feinberg F, Luo T, Hedden T, Gutchess AH (2004). A cross-culturally standardized set of pictures for younger and older adults: American and Chinese norms for name agreement, concept agreement, and familiarity.. Behavior Research Methods, Instruments, & Computers.

[36] Sirois M, Kremin H, Cohen H (2006). Picture-naming norms for Canadian French: name agreement, familiarity, visual complexity, and age of acquisition.. Behavior Research Methods.

[37] Cuetos F, Alija M (2003). Normative data and naming times for action pictures.. Behavior Research Methods, Instruments, & Computers.

[38] Cuetos F, Ellis AW, Alvarez B (1999). Naming times for the Snodgrass and Vanderwart pictures in Spanish.. Behavior Research Methods, Instruments, & Computers.

[39] Sanfeliu MC, Fernandez A (1996). A set of 254 Snodgrass Vanderwart pictures standardized for Spanish: Norms for name agreement, image agreement, familiarity, and visual complexity.. Behavior Research Methods, Instruments, & Computers.

[40] Miranda MC, Pompeia S, Bueno OF (2004). [A comparative study of norms for a 400 picture set between Brazilian and American children].. Revista Brasileira de Psiquiatria.

[41] Pompeia S, Miranda MC, Bueno OF (2003). Brazilian standardised norms for a set of pictures are comparable with those obtained internationally.. Arquivos de Neuro-Psiquiatria.

[42] Bates E, Burani C, D'Amico S, Barca L (2001). Word reading and picture naming in Italian.. Memory & Cognition.

[43] Severens E, Van Lommel S, Ratinckx E, Hartsuiker RJ (2005). Timed picture naming norms for 590 pictures in Dutch.. Acta Psychologica.

[44] Pind J, Jonsdottir H, Tryggvadottir HB, Jonsson F (2000). Icelandic norms for the Snodgrass and Vanderwart (1980) pictures: name and image agreement, familiarity, and age of acquisition.. Scandinavian Journal of Psychology.

[45] Ellis AW, Morrison CM (1998). Real age-of-acquisition effects in lexical retrieval.. Journal of Experimental Psychology: Learning, Memory, & Cognition.

[46] Barry C, Morrison CM, Ellis AW (1997). Naming the Snodgrass and Vanderwart pictures: Effect of age of acquisition, frequancy, and name agreement.. Quarterly Journal of Experimental Psychology.

[47] Vitkovitch M, Tyrrell L (1995). Sources of disagreement in object naming.. Quarterly Journal of Experimental Psychology.

[48] Goodale MA, Milner AD (1992). Separate visual pathways for perception and action.. Trends in Neuroscience.

[49] Goodale MA, Westwood DA (2004). An evolving view of duplex vision: separate but interacting cortical pathways for perception and action.. Current Opinions in Neurobiology.

[50] Filliter JH, McMullen PA, Westwood D (2005). Manipulability and living/non-living category effects on object identification.. Brain & Cognition.

[51] Laws KR, Neve C (1999). A normal' category-specific advantage for naming living things.. Neuropsychologia.

[52] McMullen PA, Purdy KS (2006). Category-specific effects on the identification of non-manipulable objects.. Brain & Cognition.

[53] Sim EJ, Kiefer M (2005). Category-related brain activity to natural categories is associated with the retrieval of visual features: Evidence from repetition effects during visual and functional judgments.. Cognitive Brain Research.

[54] Ostergaard AL, Davidoff JB (1985). Some effects of color on naming and recognition of objects.. Journal of Experimental Psychology: Learning, Memory, & Cognition.

[55] Davidoff JB, Ostergaard AL (1988). The role of colour in categorial judgements.. The Quarterly Journal of Experimental Psychology: A, Human Experimental Psychology.

[56] Brodie EE, Wallace AM, Sharrat B (1991). Effect of surface characteristics and style of production on naming and verification of pictorial stimuli.. American Journal of Psychology.

[57] Biederman I, Ju G (1988). Surface versus edge-based determinants of visual recognition.. Cognitive Psychology.

[58] Price CJ, Humphreys GW (1989). The effects of surface detail on object categorization and naming.. The Quarterly Journal of Experimental Psychology: A, Human Experimental Psychology.

[59] Fiez JA, Tranel D (1997). Standardized stimuli and procedures for investigating the retrieval of lexical and conceptual knowledge for actions.. Memory & Cognition.

[60] Bonin P, Boyer B, Meot A, Fayol M, Droit S (2004). Psycholinguistic norms for action photographs in French and their relationships with spoken and written latencies.. Behavior Research Methods, Instruments, & Computers.

[61] Lang PJ, Bradley MM, Cuthbert BN (2005). International affective picture system (IAPS): Affective ratings of pictures and instruction manual..

[62] Ekman P, Friesen WV (1976). Pictures of facial affect.

[63] Lundqvist D, Flykt A, Vhman A (1998). The Karolinska Directed Emotional Faces..

[64] Bonin P, Perret C, Méot A, Ferrand L, Mermillod M (2008). Psycholinguistic norms and face naming times for photographs of celebrities in French.. Behavior Research Methods.

[65] Viggiano MP, Vannucci M, Righi S (2004). A new standardized set of ecological pictures for experimental and clinical research on visual object processing.. Cortex.

[66] Adlington RL, Laws KR, Gale TM (2008). The Hatfield Image Test (HIT): A new picture test and norms for experimental and clinical use.. Journal of Clinical and Experimental Neuropsychology.

[67] Snodgrass JG, Corwin J (1988). Perceptual identification thresholds for 150 fragmented pictures from the Snodgrass and Vanderwart picture set.. Perceptual and Motor Skills.

[68] Cano ME, Class QA, Polich J (2008). Affective valence, stimulus attributes, and P300: Color vs. black/white and normal vs. scrambled images.. International Journal of Psychophysiology.

[69] Van Schagen I, Tamsma N, Bruggemann F, Jackson LL, Michon JA (1983). Namen en normen voor plaatjes.. Nederlands Tijdschrift Voor De Psychologie.

[70] Alario FX, Ferrand L, Laganaro M, New B, Frauenfelder UH (2004). Predictors of picture naming speed.. Behavior Research Methods, Instruments, & Computers.

[71] Stewart F, Parkin AJ, Hunkin NM (1992). Naming Impairments Following Recovery from Herpes Simplex Encephalitis: Categor y-specific?. Quarterly Journal of Experimental Psychology.

[72] Gomez P, Shutter J, Rouder JN (2008). Memory for objects in canonical and noncanonical viewpoints.. Psychonomic Bulletin & Review.

[73] Bulthoff I, Newell FN (2006). The role of familiarity in the recognition of static and dynamic objects.. Progress in Brain Research.

[74] Wurm LH, Legge GE, Isenberg LM, Luebker A (1993). Color improves object recognition in normal and low vision.. Journal of Experimental Psychology: Human Perception & Performance.

[75] Ishai A, Ungerleider LG, Martin A, Haxby JV (2000). The representation of objects in the human occipital and temporal cortex.. Journal of Cognitive Neuroscience.

[76] Ishai A, Ungerleider LG, Martin A, Schouten JL, Haxby JV (1999). Distributed representation of objects in the human ventral visual pathway.. Proceedings of the National Academy of Sciences of the United States of America.

[77] Chao LL, Martin A (2000). Representation of manipulable man-made objects in the dorsal stream.. NeuroImage.

[78] James TW, Humphrey GK, Gati JS, Menon RS, Goodale MA (2002). Differential effects of viewpoint on object-driven activation in dorsal and ventral streams.. Neuron.

[79] Kosslyn SM, Alpert NM, Thompson WL, Chabris CF, Rauch SL (1994). Identifying objects seen from different viewpoints. A PET investigation.. Brain.

[80] Snodgrass JG, Vanderwart M (1980). A standardized set of 260 pictures: norms for name agreement, image agreement, familiarity, and visual complexity.. J Exp Psychol Hum Learn.

